# New York Correspondence

**Published:** 1862-12

**Authors:** B. Wood

**Affiliations:** 1½ Clinton Place, New York


### Correspondence.

Terre Haute, Ind., November 2d, 1862.

*Editors Register:*—Having some leisure moments at my disposal, as a *new* dentist in a strange place is not unapt to have during an indefinite period of probation, I have thought to employ them for my own diversion, if not for the profit of your readers.

It was some gratification to find not only the good people of this flourishing western city, but the country population surrounding it, well educated up to the point of appreciating the importance of the care and preservation of their teeth, as well as an intelligent estimate of the claims of good dentistry and a commendable disposition to reward skill with a liberal fee. To these results, the Drs. Smith have, in a large degree contributed, and they have only been accomplished, doubtless, by long continued and patient instruction of the popular mind, and the no less effective and convincing teachings of good dentistry. For fifteen years or more, these gentlemanly practitioners have labored to elevate the profession here, and the confidence reposed in them by community, the general excellence of their operations, and the education of people in matters relating to dentistry, testify how successfully they have improved their field of labor, and how faithfully they have fulfilled their obligations to their patrons and the profession. I have, within the past few days, seen fillings inserted by Prof. H. R. Smith ten years ago that were superior operations, and have proven eminently *conservative.* Their exterior finish was not in the highest style of the art, but they were *honest* plugs to the core. With more care in finishing them, they would have made the fortune of a less modest man.

Dr. Samuel B. Smith, who continued the practice in this place, after the election of his elder brother to the chair of Mechanical Dentistry in the Ohio College, in 1853–4, has recently retired, temporarily at least, from the active duties of the profession. The Doctor is, in the highest sense of the term, an agreeable and polished gentleman, and enjoyed with singular uniformity the confidence and esteem of all who knew him. It is to be greatly regretted that one who has so worthily sustained himself in his profession, should feel constrained to abandon the practice when men of integrity and ability are so much needed to live down the pretensions of miserable incompetents. I hope he may experience the same success in his present enterprise that crowned his professional labors here.

On my way to this place, I made a brief call upon Drs. Hunt and Johnson of Indianapolis. The office appointments of these gentlemen are what might be expected of cultivated men devoted to their profession,—convenient, tasteful and complete in all their details. The large patronage which they command is evidence that their skill and accomplishments have not gone begging.

I can not omit this occasion of expressing my unqualified admiration of some superbly executed fillings by Dr. G. P. Hunt, which lately came under my notice. The reader must pardon me for indulging in these personal allusions, and the subjects of them must bear with me good-naturedly, for it is part of my religion to cheerfully credit and do homage to professional excellence or merit whenever and wherever I meet it. I am affected by a masterly piece of workmanship in the mouth as I should be on discovering a gem of rare value amidst a mass of unseemly rubbish. There is inspiration in a faultless filling fresh from the hands of a master, and I never look upon such except with feelings of deepening regard for my profession, and renewed aspirations to excel in this beautiful art. The operations referred to were proximate fillings in the superior incisors of a professional brother of Evansville, Indiana. If these fillings might be taken as an average sample of the Doctor's skill in this department, he is fairly entitled to rank as a first class operator, for I am free to say, that I have never seen them excelled in all the properties that constitute a good plug. The cavities were such that no ordinary skill could have accomplished successful results, and I thought, when examining them, that the operator, with the simple change of name, might properly have adopted the remark of a celebrated English Dentist, who, when he had completed a skillful plug, turned to his patient and said with the earnestness of conviction, "Sir, when you are an old man, and look upon that filling, your grateful exclamation will be, 'blessed be Waite.'"

Although I have not seen any work from the hands of Dr. Johnson, I am informed that he sustains an enviable reputation as a finished operator upon the teeth. We were class-mates in the Ohio Dental College during the session of 1852–3, since which time he has been steadily rising in the profession, and has made himself favorably known by his zeal in societary movements, and devotion to the elevation and interests of his calling. He enjoys a large patronage, and merits it withal. Yours, &c., J. R.

---

Mr. Editor:—Sometimes you have honored me with an invitation to write for the *Register;* probably twice or thrice as often I have resolved in the capacity of a private individual, on my own responsibility, to contribute my humble mite to the support of our current professional literature, but these resolutions, like their myriad kindred, have vanished and left no record of their existence. Will you permit a line of kindly criticism, on two articles of kindred character, by the same author, W. H. Shadoan, on fang-filling and pivoting teeth, in the July and August numbers, and before we proceed to notice the positions taken by your correspondent, permit me to ask, for information, in what class of society of almost paradisiacal innocence and purity is it the good fortune of our "brother chip" to live and labor? where the inhabitants never say, "I am sick, but only suffer an occasional twinge of docile tractable tooth-ache, which only requires the skillful interference of some genuine D. D. S. to lay it at rest forever. He sets out by asking if a tooth *is* diseased at the root why not *cure* it as well as the physician cures his patient of periostitis? Perhaps he can if the disease "at the root" is only periostitis, but suppose the periostium be already destroyed, over one fourth, one half, or even a greater extent of the surface of the fang, what then? Would he put it through a regular course of medicine, as the Thomsonians used to do their patient? Employ undoubted *skill* and when every other recourse failed, amputate. Sometimes the fang may be reached through an external opening, and the denuded surface cut away, and the irritating impurities washed out, after which the surrounding parts may assume a semi-healthy condition for a time, but the man presumes largely on the credulity of his auditors who undertakes to say that a tooth *cured* in this way is just as good as any other. But I can not undertake to notice all the inaccuracies in this article. It no doubt contains many valuable truths, truths generally known to all who seek correct information in regard to the principles of the profession, but I apprehend the tendency of the article would be to mislead those who might attempt to follow its guidance. Dr. S. may be a skillful and successful operator, but the view he takes of this subject, as a theorist, reminds me of a man looking at a distant object through a telescope; he obtains a magnified perspective of the surface directly opposite, while that of every other part, and of all surrounding objects, is entirely excluded. True, there is one saving clause in his theory, to which he makes the slightest allusion imaginable, where he says "I have cured teeth of almost all manner of disease, and under favorable" circumstances it can always be done," if he had only stopped long enough to tell us by what rule or test we may decide at sight, as to whether the "circumstances are favorable" or otherwise, in any and every case that may present itself, then he would have accomplished a work of benefaction of no small magnitude. H. McCULLUM.

*November* 1, 1862.

### NEW YORK CORRESPONDENCE, LETTER FROM DR. B. WOOD.

NEW YORK, November 17, 1862.

DR. J. TAFT, *Dear Sir*:—I sent you part of my paper on Metals and Alloys some days ago and mentioned that in a day or two I would give you an account of some improvements in the use of the Plastic Metallic Filling. These relate somewhat to the manipulation but chiefly to the adaptation of enamel or porcelain caps to the plugs.

As to the manipulation, little can be offered but what will readily suggest itself in practice. What I say therefore, is for beginners, assuming that you will publish the remarks. If you have an assistant you can expedite the operation, particularly in cases of large crown plugs, by introducing at once the quantity required in the melted state, and quickly pressing it to its place with the finger. If you have no assistant carry the metal on the blade of your plugger to the place and slip it off into the cavity with the forefinger, pressing down firmly at the same time. This drives it into the sinuosities. The finger may be covered with a cap of chamois, or of paper. The former, besides being a slow conductor, has the advantage of being soft and yielding, like the papillary surface of the finger, so as to press the filling securely into the anfractuosities of the cavity. Paper is one of the best non-conductors of heat· The metal will remain soft upon it for several moments. In this way it may be carried to the desired place more at leisure. A paper scoop or spatula may prove very convenient. Spunk is also useful as a *metal carrier*, to be borne on the end of the finger. I merely suggest these substances; practice will indicate when and how to use them.

For small cavities it is generally best to introduce at once enough metal to fill them, applying it soft and pressing it down quickly. In this way you can fill half a dozen cavities, already prepared, in as many moments, finishing off at leisure. But it requires *skillful manipulation.* And here it may be said, that, while in skillful hands the metal can in most cases be applied with as great facility as any other material whatever, it can not be used by bunglers at all so as to make good fillings. And this will prevent it ever becoming a hobby with quacks, like amalgam and oxichloride of zinc, which can be introduced about as well by the neophyte as by the proficient dentist. Those who expect to get the "knack" of using it by a few slovenly trials will soon fall back upon their "pastes." It requires care, judgment and ingenuity. Let as much pains be exercised as in introducing gold fillings until the necessary expertness is acquired, and then all will go well. When crystal gold and adhesive foil were introduced members of the profession spent infinite pains to become proficient, without balking at the expense of the material. They were not content to make "trial" of enough to fill two or three teeth and upon that to pronounce judgment. With the same zeal and spirit we should fairly test the merits and demerits of any new material that promises to be of real advantage to the profession.

In building out broken teeth with the Plastic Metallic Filling, leave the healthy fragments, as they serve to give support. When the nerve cavity is opened silver or platina pins may be made to project from them for the same purpose. But the operator should be particular that the fangs are perfectly healthy in such cases, for if trouble occurs the patient is sure to lay the fault to the material, if it is anything but gold, and particularly if it is a new material.

For pivoting, unless you use an interior wire, it is well to cut a lateral mortice-shaped groove, with the file, bur, or chisel, as it gives additional stability and strength, the apex of the root being filled with foil, or a non-conductor, as the metal chills before it can be introduced perfectly into an orifice so small and deep.

But to come to the improvement by means of enamel or porcelain caps, of which I designed more especially to speak.

This has been maturing for some time, although matters more immedially pressing upon attention have impeded progress.

The design of this improvement is to restore the form and beauty of decayed and broken teeth, at the same time preserving as much as possible of the healthy dentine, and also to conceal the metallic plugs, by means of a cap or covering resembling the sound parts of the tooth. The appliances may be wrought out of ivory, dentine, or other substances, but the design for the most part is to have them prepared ready for use, of a variety of forms and sizes, out of the material used for making artificial teeth.

These caps or appliances are from the size of a medium large plug to that of the entire crown of a tooth having slots, cavities, or asperities to serve as retaining points for the filling. For the molars and bicuspids I make wedge-form slots, or cavities resembling in form the nerve cavity, and sometimes orifices penetrating the coronal fossæ. Where one half or one third of the tooth is gone the caps to be used are one half or one third crowns, with slots or cavities as indicated. These are *plugged* with the Plastic Metallic Filling, and then applied, hot enough to soften the metal to the plugs built up with the same material on the natural teeth in the proper form to receive them.

In this way entire crowns may be engrafted upon the remains of teeth—back as well as front—and *without removing living nerves,* as is done in excising and pivoting teeth according to the ordinary plan. The advantage of this improvement in this particular alone is too obvious to dwell upon. It saves the shock of excising the dense dentine, the torture of exterpating the nerve and the mischief inflicted in the destruction of this life-giving principle.

Where the caps are for anterior surfaces, as in cases of those cavities we so often find on the labial surface of the incisors and cuspidati near the gum, they are formed with notches or grooves on the sides.

I have assumed the Plastic Metallic Filling as the material to be used for engrafting the cap to the natural base, but it will occur that any plastic material possessing sufficient tenacity, and otherwise suitable, would answer the purpose. It will also occur that caps may be formed of thin gold or platina plate and applied in like manner to plugs built up with the metallic filling upon fragments of teeth.

It was my intention to have taken steps, by filing a caveat until I should have reduced the process to its most practical form, to secure Letters Patent for this improvement. (I intended to do so chiefly by way of taking ground on the side of a great principle unjustly assailed.) But the pressure of other matters renders it uncertain what time I should have to devote to it, and I do not want to keep the profession in suspense. Besides, I am anxious to have as many co-laborers as possible in this direction.

As to patents. There are always those who stand ready to appropriate any discovery or improvement of pecuniary value, and twist it into such form as to obtain a patent upon it, (excluding the real discover from the fruits of his labor) and exact an unreasonable fee for "licenses." If the thing can not be appropriated in this way, there are enough to claim priority of discovery, and keep the matter in perpetual wrangle. But if the inventor patents his invention it puts a stop to this. Others indeed may put in claim for the honor, but they can invalidate his patent by making good their pretensions, and that they do not is conclusive on the point.

But the principle of patenting is right in itself. It is preeminently wise and good. It has been found by the ablest statesmen and philosophers of all enlightened nations a most efficient means for the advancement of the arts and sciences, and for promoting the progress and well being of humanity. When therefore, we see combinations of men in our own profession whether influenced by mistaken zeal, by ambition for notority, or in mere passive compliance at the behest of uneasy spirits of stronger frames than their own arraying themselves against a principle which has blest the world, it becomes us to stand up against the wrong. Better encourage invention in our profession by every available means. Better lend the helping hand to the struggling inventor and provide him, if need be, with means to secure him the fruits of his labors, than to throw odium upon him, harass him with litigation and so drive him from the field of usefulness, to make room for that other class who are able enough to bear these things. What we need to call into exercise is the spirit and intelligence to *examine*,—not a childish touchstone, such as whether a thing is "patented" or not!

In regard to the Plastic Metallic Filling, I have been disposed for the present to leave it altogether with the profession, and have therefore as yet made no effort to introduce it myself, except the simple announcement. I have not undertaken to present its claims to the public, to dental societies, nor to individual members of the profession; only at a meeting of the "New York and Brooklyn Dental Society" held at my office severial nights ago, I exhibited at their request some specimens of plugging and pivoting and the mode of using it. I understand that a number of our best operators are giving it a trial, (I know of several) and we shall no doubt hear the results of their experience in due time. So far as I have yet heard an opinion it has been decidedly favorable.

I have been anxious that the better class of dentists should acquire some skill in its manipulation before it passed into general use. It will give them the advantage of prior proficiency, at the same time instructing the community, through them, as to the manner of using it and to discriminate between its skillful and unskillful use. To have been over-earnest to push it would probably have thrown it at first mostly in the hands of bunglers and thus prejudiced the community, which would have put it back far beyond what is lost by a little delay. But all reading men in the profession, who are looked up to as posted, have had time enough, so that I ought not to hold back now.

As to its merits, it need not take long to determine. It shows precisely what it is. As a substitute for gold it was not offered. As a substitute for any other form of the baser metals or compounds now employed, I believe it has advantages over any or all of them. No one can put it to severer tests than I have done: and no one I think can urge any valid objection not fairly stated or suggested by me in the paper through which I first announced it to the profession.

Of the state of the profession in the city I can say little from personal observation, not having had time to look abroad much among the dentists, but those whose acquaintance I have made at the societies have impressed me most favorable as to the status of the profession among the better class. There are two societies in the city, the "New York and Brooklyn Association" and the "New York Society." The former hold their meetings at the residences of members, the latter at the Cooper Institute building. They are carried on with spirit and general good feeling. Papers are read and discussed, instruments exhibited, and the members seem all animated by an intelligent zeal for advancement. To the profession in this city, these societies, if properly sustained, will prove the leaven of regeneration. They serve to counteract that retrograde tendency which the isolation, self sufficiency, and apathy, into which individual members are so prone to lapse in a great city like this, superinduce; arousing all to a sense of deficiency and of the necessity of continued effort, no matter *where* they happen to *reside.*

There is another society, or combination, which appears to have been gotten up for a different object, turning aside from the legitimate objects of the science in order to pursue and combat patents. If simply to expose impositions claiming the respectability of patents it will hardly pay the amunition, ending most likely like the "amalgam war;" with a temporary notoriety for the belligerents on both sides, and nothing further. If it is to worry inventors and damage their rights and interests by litigation, it may be well to admonish the unsophisticated that the law is pretty heavy upon "*combinations* against the rights and interests of individuals.

A birds-eye view, judging from the majority of those who claim to be dentists in the city, would impress one rather unfavorably. Indeed it would strike him as the elysium of charlatans. The most wiley means are used to decoy customers, and the most villainous expedients to swindle them. One advertises to fill with gold for fifty cents, and when his customer comes, he perhaps fills one or two loosely, and then pries between and fumbles about sound teeth with his instrument and a fragment of foil, says he has filled them, gets his fee, and *boasts of it afterwards.* A lady calls upon another to have her teeth filled with "platina," (some one is advertising "white platina" fillings.) "Certainly, ma'am," the operator "uses it extensively," so he fills them with *amalgam,* at a high rate, and then brags over the fraud.

Some having devices in their show windows to outwit the "devices of the devil"

"When he throws out decoys for prey,
Poor mortal souls suborning,"

at a cost that would support half a dozen honest dentists and families. Revolving wheels with all sorts of tooth "fixin's." Wax figures in motion, men and women, as large as life. At one moment a handkerchief or bouquet goes up to the mouth, it is withdrawn and reveals a horrid grin of black distorted *stubs.* It goes slowly up to the lips again, and, on being withdrawn the next moment, discloses a set of white *teeth* yet so ghastly that one really regards the first "show" as the more agreeable. These things "take" much money out of wonder-gaping pockets. "So wags the world" of New York.

There is some old foggyism among the better class of dentists. They think they live in *New York* and *consequently* know every thing, so *consequently* they lay behind the general progress of their class. They love the beaten track, and when constrained by outside pressure to leave it, yet "turn one last lingering look behind." The mallet has found exceedingly few here to give it even a trial.

The truth is, New York is the best place in the world to remain in *statu quo* unless one is constantly pushing his *antennea* outside. Business of every kind is so much divided, that one has to run to a different place for every little thing, and, if up town, perhaps two or three miles at that, (and a ride down town for a few trifles consumes the best of half a day.) In like manner you have to inquire hither and thither for any little information. No one knows anything apart from his particular calling, nor seems to care. The dentist who does not cast abroad here and there in quest of information (or at least take the journals—that pay attention to any new thing save their special hobbies,) may, after a life time, die here in the knowledge with which he started. In small towns all know something about general matters. It may be but a smattering, but it suffices to arouse attention; and each fragment of information affords a clue to the ingenious mind towards the right track. In such places the dentist can not remain totally unconscious of the improvements in his profession; the whole community serving as prompters, "coerce" him to post himself up and to adopt what is useful, or lose caste.

But our societies, if they do not fall out among themselves, or fly off the right path after some "Will o' the Wisp," will operate upon the huge mass and quicken it into life after a while.

Now I have written you a long letter, such as it is. I presume you will publish that part relating to my improvement. I dont know that there is any thing else said that would interest the profession. If there should be, you can make use of it as you choose.

I will send the paper on metals, &c., for your next, and should in the meantime be glad to hear what is doing in Cincinnati and also what defects you find in the Plastic Metallic Filling, that I might endeavor to obviate them.

Yours very respectfully,

1½ Clinton Place, B. WOOD.
New York.

